# Euthanasia

**Published:** 1897-04

**Authors:** 


### Euthanasia.

"When time, or soon or late, shall bring
The dreamless sleep that lulls the dead,
Oblivion! may thy languid wing
Wave gently o'er my dying-bed!"

So wrote Byron in contemplation of the end of his wretched life. The wish that one may "painlessly attain the end of pain" is as old as the reflecting mind of man, and modern medicine is certainly advancing toward a point which might make the wish an attainment.

For not only are surgical operations rendered painless by anesthetics, but acute painful diseases are made endurable, and death itself is well nigh robbed of its terrors by the "respite and nepenthe" of the skillful therapeutist.

So far modern medicine has shown an ability to deal with the problems of surgery and acute disease which would place it above the criticism of Socrates. But in dealing with the victims of chronic and incurable diseases the physician of to-day is almost as helpless as were the Asclepiadæ of the philosopher's era.

In the opinion of Socrates medicine and surgery should be applied to such cases as were curable, it being ridiculous "to pay so much attention to regimen and diet as to drag on a miserable existence as an invalid in the doctor's hands. . . . Esculapius, it was maintained, revealed the healing art for the benefit of those whose constitutions were naturally sound; he expelled their disorders by drugs and the use of the knife, without interfering with their usual avocations; but when he found they were hopelessly incurable, he would not attempt to prolong a miserable life by rules and diet, as such persons would be of no use either to themselves or the State. Constitutionally diseased persons and the intemperate livers were to be left to be dealt with by nature, so that they might die of their diseases."

This method of neglecting the chronically diseased and incurable is in perfect keeping with the economic practice among the Greeks of casting on the rocks all bantlings who, in consequence of mal-development or inherited disease, were not thought fit to survive. These ideas, of course, are heathen, and by no means in accord with the system of Christian charity under which we live, but they were nevertheless expedient, and with modern appliances for softening the method of extermination might not be cruel, and perhaps not unmerciful.

Indeed, one of the arraignments which a strict application of philosophic principles must make against medicine to-day is that it promotes in much of its function the survival of the unfit. In too much care for the single life it forgets to be careful of the type, and in consequence the hereditarily diseased are kept alive until they have time to reproduce themselves, and thus perpetuate their infirmities through coming generations. Of course the Greek method of remedying the evil is not to be thought of, but certainly some wise restricting legal measures ought to be taken against the transmission of hereditary disease through marriage.

A natural and logical extension of this question is euthanasia for the relief of persons suffering with painful and necessarily fatal diseases. It has incidentally come to the surface several times during our quarter of the century, but seems to have enlisted only a few timid advocates on the one hand, with a host of vehement antagonists upon the other. A recent letter to the *New York Journal*, by the Rev. Charles W. Wendte, sets forth the question logically, cogently, and without offense. He says:

"Although I strongly advocate the destruction of certain people by humane methods, I am not willing that my position in this matter should be misunderstood. All pioneers of thought have suffered misrepresentation, and I desire, as much for the benefit which may result to the world as for the defence of my own reputation as a humane man, to be thoroughly comprehended.

"To my mind one of the most deplorable and moving sights in Christendom to-day is the condition of the incurably diseased among us, especially if they are also in impoverished circumstances. Society should do all in its power to relieve this class, alleviating by all the resources known to modern medical science, and placing them above the mental distress and physical misery attaching to personal want.

"But who is to take the responsibility? Certainly not the medical man. On the field of battle some latitude may be allowable to him, but in the ordinary course of life no single individual is to be intrusted with so grave a responsibility. Shall we, then, continue as now, powerless to relieve the fearful suffering of so many unfortunates, turning a deaf ear to their entreaties and leaving them but one resource—suicide? In the name of humanity I say, No!

"A few years ago an engineer of eminence in France, afflicted with a terrible inherited disease, of which he had seen his own father perish under aggravated tortures, took his own life. In his will he explained the violence of his deed, and left to the French government a large bequest for the establishment of a commission on euthanasia. The French government, it was claimed, by clerical counsels, declined to accept the trust.

"In this, I believe, it did wrong. Though I appreciate the difficulties attending the matter, and what special dangers to society it may seem to carry with it, I yet believe that the time is fast approaching when the painless destruction of incurables will be considered eminently wise, humane and Christian.

"As in the case of the French suicide, my theory meets with the greatest opposition in clerical circles. In the first place, I am told that He who gives life should take it. Now, I hold that in that case all capital punishment, all forcible resistance to personal violence which may cause death, all war, either offensive or defensive, is wicked and can not be justified. The murderer should go free, the assassin should go unhanged, and the greatest generals, in place of being lauded as patriots, should be condemned as malefactors.

"It is plain therefore that this doctrine is untenable, and is constantly and rightly ignored by society. To go further, look at the thousands of diseased, suffering, tortured human beings who crawl on the surface of this fair earth—the offspring of human ignorance, brutality, guilt, and shame. Surely, if justice and humanity were to decide the matter, such unfortunate beings would never be allowed to come into existence at all. In fact, leading physicians have told me that they never allowed a human monstrosity to live after it was born.

"But we ought to go a step further. It ought to be a universal law throughout Christendom that the child-bearing of idiots, criminals, the insane and all incurably diseased persons should be prevented. How? By making it necessary that the physician's certificate should precede the issuance of the marriage license. Go into the children's hospital and see the little innocents, malformed, racked. and tortured from no fault of their own. Afflicted with terrible wasting diseases, without the possibility of permanent restoration, or even relief, death, in many cases, is only a question of weeks or months. Why insist on continuing the tortures so needless, so undeserved? Do my antagonists think that God loves to behold the agony of infants?

"Another thing which my clerical critics assert is that God sends pain to discipline and purify us, and we ought not to resist his will. In that case the discoverers of ether and chloroform were guilty of great impiety, and the monument to their memory in Boston should be pulled down.

"Now I argue, as a general thing, that pain—excessive pain, I mean—does not purity the spirit of man. On the contrary, it blunts, hardens, and brutalizes it. Of course there are noble exceptions which go to prove this rule. By our present methods we drive men to suicide by our inhumanity toward them. A pitiful case in point came under my own observation.

"Now, in order to put a check to such happenings, to put behind us all sickly, false sentimentality in the handling of cases such as I have cited, I suggest that there should be formed a properly constituted tribunal, acting only when duly called upon by suffering humanity, and then under every necessary safeguard and restraint. Let this jury consist of a number of medical men, representatives of the government, and any others who might advantageously serve. To this tribunal have submitted any petition for examination and release which might be made by the incurably diseased and indorsed by their families. Then, if they were found incapable of recovery and sure to endure needless and great agony, the tribunal shall be empowered to gently, painlessly and humanely put them out of suffering and give them a release into the better world. This, then, is my plain. The surgeon must give pain to effect a cure. So qumankind, which, under God, gives life, may, in the divine spirit of justice and mercy, take it back again.

"It may be urged that this theory is of pagan and not Christian origin. What of that? I am discussing its wisdom, not its origin. To lessen the sum of human misery in the world should be our chief endeavor as humanitarians, regardless of creeds, and the calm discussion of euthanasia, which is now in progress, encourages me that under certain plain restrictions it will inevitably be adopted by all civilized and Christian nations."

The least that could be urged on this terrible question is the advisability of allowing a wretch whose sufferings have driven him to suicide to have his quietus made by the authority of the State, instead of thrusting himself into the unknown with the stain of self-murder upon his soul.—*American Practitioner and News, Nov. 28, 1896.*

---

**Resorcin in the Treatment of Eczema.**—Dr. Hartzelel (cited in the *Independence Medicale*, Jan. 6, 1897) recommends the following formula for erythematous eczema:

℞ Resorcin............................7½ grains.

Glycerin............................30 grains.

Limewater..........................450 grains.

M. Sig.: To be applied for a few minutes several times a day. This is said to allay the itching and check the secretion rapidly. After that, he employs the following:

℞ Vaseline.............................300 grains.
Powdered starch, } each......75 grains.
Zinc oxide, }
Resorcin............................15 grains.

M. Make an ointment, to be used two or three times a day. —*New York Medical Journal.*

**The Intelligent Compositor.**—Ours is a marvel. We wrote: "Is there no balm in Gilead?" Supposing we didn't mean it, or were mistaken in the town, our compositor slung it up "Goliad." But that's a small matter. Some years ago, in compiling a mortuary report from exchanges, we wrote: "Dr. Jno. B. Bailyhache, *œt* 74 years, born July 20, 1822, and died —." Imagine our consternation on finding the proof sheets to read: "Dr. Jno. Bellyache eat 74 years corn July 20, 1822, and died—."

---

**A New Departure in Essays.**—The JOURNAL has been favored by the author with a copy of his "Essay on the Management of the New-born Babies," by Dr. T. Y. Lewis, of Dublin, Tex. It is accompanied with the announcement that "At the monthly meeting of the Medical Association of Erath county, Texas, held at Lingleville, Tex., September 1st, 1896, Dr. T. Y. Lewis, of Dublin, Tex., was appointed to prepare an essay on the best management of new-born babies, to be read at the next monthly meeting, to be held in Stephensville, Tex., October 6, 1896. After hearing the essay read the Association voted to have it printed."

The copy sent us is written and printed in the ballad style, a form much affected by the illustrious author of "Fare you well my trim-built wherry," a "literary gent *with* a wooden leg."

We cannot reproduce the essay, much to our regret, and to the eternal loss of our readers, but we give just a few couplets, to show the talented author's style, which, we must say, is unique. The doctor leaves the beaten track, making "a new departure," he says; thus:—

"I did not consult books or medical journal—
For these few lines,—as they came up internal."

After some preliminary remarks, the doctor pitches in, *in medias res*, and says,—the baby being born,—

"The girdle they put on him, round about,
Is to keep his navel from bulging out."

The author announces one truth, especially, which will find a cordial echo in the hearts of his readers:

"It is not so hard to look after the baby
As it is to watch some meddlesome old lady."

The Erath County Medical Society is to be congratulated on the possession of so sweet a singer. Our medical poets will have to look to their laurels.

